# DNase SISPA-Next Generation Sequencing Confirms Schmallenberg Virus in Belgian Field Samples and Identifies Genetic Variation in Europe

**DOI:** 10.1371/journal.pone.0041967

**Published:** 2012-07-27

**Authors:** Toon Rosseel, Matthias Scheuch, Dirk Höper, Nick De Regge, Ann Brigitte Caij, Frank Vandenbussche, Steven Van Borm

**Affiliations:** 1 Virological Platform Unit (VIRPLAT), Operational Direction Viral Diseases, Veterinary and Agrochemical Research Center, Brussels, Belgium; 2 Institute of Diagnostic Virology, Friedrich-Loeffler-Institut, Greifswald-Insel Riems, Germany; 3 Unit of Enzootic and (Re)emerging Viral Diseases (ENZOREM), Operational Direction Viral Diseases, Veterinary and Agrochemical Research Center, Brussels, Belgium; Institute for Animal Health, United Kingdom

## Abstract

In 2011, a novel Orthobunyavirus was identified in cattle and sheep in Germany and the Netherlands. This virus was named Schmallenberg virus (SBV). Later, presence of the virus was confirmed using real time RT-PCR in cases of congenital malformations of bovines and ovines in several European countries, including Belgium. In the absence of specific sequencing protocols for this novel virus we confirmed its presence in RT-qPCR positive field samples using DNase SISPA-next generation sequencing (NGS), a virus discovery method based on random amplification and next generation sequencing. An in vitro transcribed RNA was used to construct a standard curve allowing the quantification of viral RNA in the field samples. Two field samples of aborted lambs containing 7.66 and 7.64 log_10_ RNA copies per µL total RNA allowed unambiguous identification of SBV. One sample yielded 192 SBV reads covering about 81% of the L segment, 56% of the M segment and 13% of the S segment. The other sample resulted in 8 reads distributed over the L and M segments. Three weak positive field samples (one from an aborted calf, two from aborted lambs) containing virus quantities equivalent to 4.27–4.89 log_10_ RNA copies per µL did not allow identification using DNase SISPA-NGS. This partial sequence information was compared to the whole genome sequence of SBV isolated from bovines in Germany, identifying several sequence differences. The applied viral discovery method allowed the confirmation of SBV in RT-qPCR positive brain samples. However, the failure to confirm SBV in weak PCR-positive samples illustrates the importance of the selection of properly targeted and fresh field samples in any virus discovery method. The partial sequences derived from the field samples showed several differences compared to the sequences from bovines in Germany, indicating sequence divergence within the epidemic.

## Introduction

During the summer and autumn of 2011, a novel disease with symptoms including fever, decreased milk production and diarrhea, was identified in dairy cattle in Germany and The Netherlands [1], [2]. Using a metagenomic analysis on next generation sequence data produced from the blood of symptomatic animals, a novel Orthobunyavirus was shown to be associated with the disease [1]. The virus was preliminary named Schmallenberg virus (SBV) according to the geographical location of the index case. A specific real time RT-PCR test was developed, confirming the presence of the virus in diseased bovines. Animal experiments with the isolated virus further supported a causal relationship between the virus and the disease [1]. In addition, the virus proved to be associated with an outbreak of congenital malformations and abortions in both ovine and bovine [1], [3], [4]. The dissemination of real time RT-PCR protocols to laboratories throughout Europe allowed the detection of Schmallenberg virus in six additional countries, including Belgium, France, Luxembourg, United Kingdom, Italy, and Spain [5] and provided evidence for the involvement of *Culicoides* sp. midges as possible vectors [6].

In the absence of targeted sequencing protocols for this novel virus, we applied a virus discovery strategy based on random amplification of purified nucleic acids in combination with next generation sequencing on SBV real time RT-PCR positive tissue samples to confirm the presence of SBV and obtain preliminary sequence data on SBV from Belgium. We previously validated this DNase SISPA (Sequence Independent Single Primer Amplification, [7]) approach on other RNA viruses [8], [9]. It consists of a viral nucleic acid enrichment step (size selective filtration in combination with a nuclease treatment to remove nucleic acids that are not encapsidated in virions) followed by a random cDNA synthesis and amplification step. The random amplicons are subsequently exploited by next generation sequencing [10]. The combination with quantitative real time RT-PCR results from the field tissue samples allowed a first estimate of the sensitivity of this approach using field samples infected with an emerging disease.

## Results and Discussion

**Figure 1:**
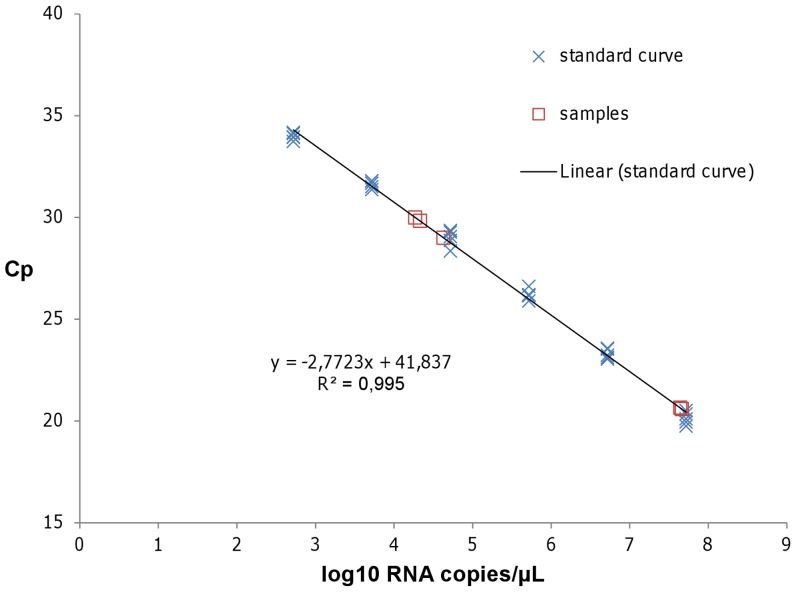
Quantification of Schmallenberg virus L segment RNA using quantitative real time RT-PCR. The viral RNA load in field samples was quantified with real time RT-PCR using a standard curve consisting of in vitro transcribed L segment RNA spanning the diagnostic RT-qPCR, which was run in five replicates (blue crosses). The linear trendline and the associated standard curve equation are displayed. The samples are indicated by red squares.

To test the feasibility of virus identification using DNase SISPA-next generation sequencing (NGS) and to get a first estimate of its sensitivity on field samples, we selected both strong and weak positive SBV infected field samples. For viral RNA quantification, in vitro transcribed RNA was used as a standard curve in the previously described L gene real time RT-PCR. The curve showed a linear range at least from 2.75 to 7.75 log_10_ RNA copies per µL, and a sensitivity of less than 2.75 log_10_ RNA copies per µL (Figure 1).

**Figure 2:**
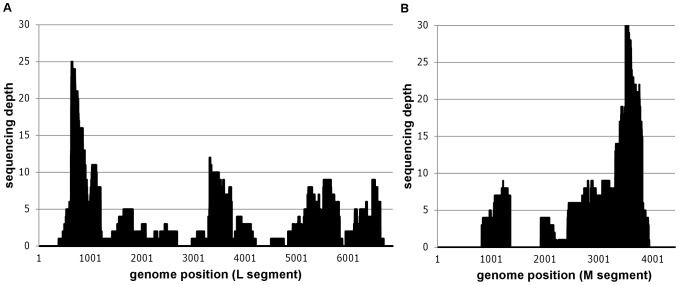
Mapping of Schmallenberg virus specific reads of sample BE/12-2479 against the German isolate BH80/11-4. Positional sequence coverage (number of sequence reads for given nucleotide position) of the M and L segments of sample BE/12-2478, based on reference assembly to HE649912 and HE649913.

Two field samples of aborted lambs with Cp values of 20.59 and 20.65 corresponding to 7.66 and 7.64 log_10_ RNA copies per µL (Table 1) allowed unambiguous identification of Schmallenberg virus. One sample (BE/12-2478) yielded 2 S segment sequences (covering about 13% of the S segment), 81 M segment sequences (covering about 56% of the M segment) and 109 L segment sequences (covering about 81% of the L segment) (Table 1, Figure 2). The other strong positive sample (BE/12-2068) resulted in a total of 8 SBV specific sequence reads distributed over the L and M genomic segments. This difference in sequence coverage for two samples with a comparable SBV RNA load is most likely due to the difference in the amount of raw sequence data (Table 1). While the sequencing of BE/12-2478 yielded about 95000 reads, BE/12-2068 only resulted in circa 25000 reads probably due to DNA library quantification issues at the sequencing facility. Moreover, more tissue sample was available for DNase SISPA protocol from sample BE/12-2478 (Table 1), although the ratio of viral reads to total raw reads was superior (0.01) in sample BE/12-2068 compared to sample BE/12-2478 (0.002 ; Table 2).

**Table 1:** SBV virus quantification and confirmation by DNase SISPA-NGS in selected field samples from Belgium.

Sample (location, date)	Description	SBV log10 RNA copies/µl (Cp value)	Total no. reads	No. SBV reads per segment (% of RNA segment covered)		
				S	M	L
BE/12-2068 (Ghoy, 13.01.2012)	Brain tissue, 180 mg, aborted lamb	7,664 (20.59)	25701	-	1 (8.59)	6 (8.69)
BE/12-2478 (Deinze, 18.01.2012)	Brain tissue, 1000 mg, aborted lamb	7,642 (20.65)	94722	2 (13.68)	81(59.1)	109 (81.9)
BE/12-2649 (Reningelst, 22.01.2012)	Brain tissue, 1130 mg, aborted lamb	4,331 (29.83)	50308	-	-	-
BE/12-1235 (Sivry, 06.01.2012)	Brain tissue homogenate, 1.8 ml, aborted calf	4,630 (29.00)	27979	-	-	-
BE/12-3610 (Izenberge, 28.01.2012)	Brain tissue, 620 mg, aborted lamb	4,270 (30.00)	98648	-	-	-
Isolate BE/12-2068 (Ghoy, 13.01.2012)	Tissue culture supernatant. 5 log_10_ TCID50/ml	8,418 (18.50)	nd	nd	nd	nd

**Table 2:** Output of the metagenomic analysis on raw sequence data from the sequencing libraries from SBV-positive samples.

Sample	Total no. reads	Reads withprimer tag identification	No. reads classified into superkingdom				No. unclassified reads
			Eukaryota	Archaea	Bacteria	Viruses	
BE/12-2068	25701	23370	2270	5	15543	284 (Phycodnaviridae, Myoviridae, Siphoviridae, Podoviridae, Bunyaviridae, Mimiviridae)	3977
BE/12-2478	94722	86178	36690	11	36106	167 (Myoviridae, Siphoviridae, Podoviridae, Bunyaviridae)	5543
BE/12-2649	50308	46181	6730	-	36044	8 (Siphoviridae, Podoviridae, Mimiviridae)	2468
BE/12-1235	27979	26214	13216	5	1789	1 (Siphoviridae)	9812
BE/12-3610	98648	91458	12403	13	69057	15 (Myoviridae, Podoviridae,)	5053

Three weak positive field samples (one from an aborted calf, two from aborted lambs) containing virus quantities equivalent to 4.27–4.63 log_10_ RNA copies per µL did not allow identification using DNase SISPA-NGS (Table 1). This is consistent with an approximate sensitivity of 10^4^–10^6^ virions per ml estimated in previous studies using in vitro virus dilutions [11] or tissue biopsy samples [12]. However, it should be noted that the exact ratio between viral RNA quantities and intact virion quantities in field samples (the functional unit being detected in this method) remains to be determined. Our preliminary data indicate that RNA extracted from an SBV isolate containing 10^5^ TCID50/ml may contain up to 8.46 log_10_ RNA copies per µL (Table 1). This indicates that precaution should be taken in interpreting RNA quantities in terms of DNase SISPA-NGS sensitivity, which is determined by the amount of viral nucleic acids that remain protected in intact virions during nuclease treatment. Moreover, a comparison of approximate sensitivity with other viral discovery methods is almost impossible, as the utilized sequencing effort varies from a few 100 Sanger sequencing reads [11], [13] to about 30 million Illumina GAII reads [12]; and different sample types (targeted tissue selection, freshness of the sample) may result in different levels of host and contaminating nucleic acids. Given the limited approximate sensitivity of DNase SISPA-NGS, as any virus discovery method, careful selection of properly targeted and fresh field samples is necessary.

As expected in any metagenomic approach, a considerable part of the sequence reads represented diverse bacterial species and host nucleic acids (Table 2). It should be noted that the field samples were stored for a considerable time before the pretreatment and RNA extraction, during which opportunistic bacteria probably grew in the samples. Although we use a 0.22 µM filter to remove remaining cell fragments and bacteria, nucleic acids from disrupted cells can pass through the filter.

Other viral reads could be mainly identified as bacteriophages belonging to the families *Myoviridae*, *Siphoviridae* and *Podoviridae* (Table 2). Three reads showed partial similarity to a virus belonging to the *Phycodnaviridae* and two reads showed some similarity to viruses of the *Mimiviridae* family. None of these viruses have relatives known to infect animals. These sequences most likely represent contamination of the tissue samples during storage until analysis.

Compared to the metagenomic approach used by Hoffmann and colleagues [1] that initially identified this novel Orthobunyavirus by shotgun sequencing of total RNA extracted from clinical samples, our virus discovery protocol attempts an enrichment in viral nucleic acids by selective filtration and nuclease treatment. A direct comparison between both data sets is impossible as we treated limited amounts of tissue samples representing a different host species. Moreover, the sequencing effort per library was not identical. Both studies indicate the need of high sequence throughput and proper sample selection as critical factors for successful virus discovery using metagenomics.

The partial SBV sequence info we obtained was compared to the whole genome sequence that was determined from the virus originally isolated from diseased bovines in Germany. Several coding and noncoding mutations could be observed (Table 3). The partial data from the two Belgian ovine field samples showed together 16 nucleotide differences (of which 9 well-supported by the sequence data, Table 3) corresponding to 9 amino acid differences (of which 5 well-supported). Although this can be expected for an RNA virus that has now shown a distribution throughout a large part of Western Europe, this is to our knowledge the first documentation of genetic diversity within the Schmallenberg virus outbreak. Based upon the 8134 nucleotides in common between our partial sequence (BE/12/2478) and the genome of the virus isolated from diseased bovine in Germany (accession codes HE649912-HE649914), a mutation frequency of 1.7 10^−3^ mutations per site was observed. The time frame between the two samples was about three months and the geographical distance between the sampling sites roughly 325 km. Although only based on a three month period, the observed mutation frequency is within the documented range of Bunyavirus variability (10^−2^ to 10^−4^ mutations/site/year documented for Hantavirus ; [14]) and within the documented range of RNA virus variability (10^−3^ to 10^−4^ mutations/site/year ; [15], [16]). It should be noted that, while the samples in Germany were taken from acutely infected bovines, the ovine samples from Belgium present aborted lambs, making an estimation of the infection time of the maternal animal impossible. Future targeted molecular epidemiological studies including samples from the complete geographic range of the virus may shed light on the origin and time of introduction of this novel virus in Europe.

**Table 3:** Differences observed in Belgian SBV sequences in comparison with the German genome sequence of isolate BH80/11-4.

Strain, genome segment	Covered regions*	Number of reads	Differences observed in Belgian sequences compared to the genome of the German isolate BH80/11-4			
			Nucleic acid	Amino acid	Depth	Support°
BE/12-2068, M segment	1095–1456	1				
BE/12-2068, L segment	1271–1426	2				
	3313–3744	4	G 3490 C	E 1159 Q	4	Low
			G 3637 A	A 1208 T	4	Low
BE/12-2478, S segment	428–467	1				
	669–733	1				
BE/12-2478, M segment	823–1354	12	G 836 A	S 275 S	3	Low
			A 983 G	T 324 T	5	High
			C 998 T	F 329 F	4	High
			A 1041 G	K 344 E	4	High
			T 1201 C	F 397 S	8	High
	1920–2211	4	G 1930 A	R 640 Q	4	Low
			A 1969 T	Q 653 L	4	Low
	2248–2390	1				
	2412–3935	64	A 3558 G	N 1183 D	29	High
BE/12-2478, L segment	383–1844	44	G 1017 A	E 334 E	9	High
	1873–2682	6				
	2966–3744	19	C 3097 T	H 1028 Y	2	High
	3770–4209	4	C 3937 T	H 1308 Y	3	High
	4502–4754	1				
	4835–5888	21	T 5736 A	P 1907 P	6	High
	5950–6690	13	C 6045 T	P 2010 P	2	Low
			T 6156 C	F 2047 F	5	Low

* the indicated position is relative to the position of the used reference sequence: Schmallenberg virus, isolate BH80/11-4 (Genbank: HE649912, HE649913, HE649914).

° assessment based on low/high depth, single/double orientation of the reads, equal/different starting and end position of the reads, and quality of reads.

Our data show that DNase SISPA-NGS viral discovery technology can be used on limited amounts of field tissue samples to identify emerging diseases. However, the sensitivity of the method seems to limit its applicability to samples containing about 10^4^ to 10^6^ virions per ml. Consequently, when applying this methodology to a cluster of cases of an undiagnosed disease, it is important to select properly targeted and fresh samples as well as to test multiple diseased animals to allow correct identification of an associated virus.

## Materials and Methods

### Samples

Diagnostic field samples from suspected cases of SBV related congenital malformations in lambs and calves were selected based on their geographical location (Table 1) and on the Cp values of the RT-qPCR detecting the L-segment of the virus that was used for diagnosis [1]. This study was conducted under the authorization and supervision of the Bioethics Committee at the Veterinary and Agrochemical Research Center (VAR), following national and European regulations.

### Viral RNA quantification

Briefly, approximately 0.5 cm^3^ of brain tissue was added to 1 ml PBS and homogenized (2 min, 25 Hz) in a TissueLyser (Qiagen, Venlo, The Netherlands). The RNA was extracted using the RNeasy minikit (Qiagen) following manufacturer instructions and eluted in 50 µl. 2 µl of this RNA mixture was further analyzed by a one-step PCR using the LightCycler 480 RNA Master Hydrolysis Probes kit (Roche Diagnostics, Vilvoorde, Belgium) on a LightCycler 480 Real-time PCR system following manufacturer instructions. Primers and probe sequences for the SBV L segment detection were kindly provided by Dr. B. Hoffmann (FLI, Germany, available on request) and used at a final concentration of 1 and 0.1875 µM respectively. Two highly positive brain tissue samples (Cp<21) from aborted lambs were selected for confirmation by DNase SISPA-NGS. In addition, two weak positive brain tissue samples from lambs and one weak positive sample form a calf were selected (Cp>28). The viral RNA load in RNA directly extracted from these samples was quantified in the above described real time RT-PCR using a standard curve consisting of in vitro transcribed L segment RNA spanning the diagnostic RT-qPCR, which was run in five replicates. The in vitro transcribed RNA was independently quantified using three different approaches: NanoDrop Spectrophotometer (Nanodrop Technologies, Wilmington DE, USA), Qubit® RNA assay kit on Qubit® fluorometer (Invitrogen-Life Technologies, Gent, Belgium), and RNA 6000 Pico chip on the Agilent 2100 Bioanalyser (Agilent Technologies, Diegem, Belgium).

### DNase SISPA and 454 sequencing

The maximum available quantity of the limited tissue samples (<1,5 g; Table 1) was homogenized in about 1500 µL PBS per g of tissue using gentle homogenization in a TissueLyser (Qiagen). Sample pretreatment and SISPA was largely performed as previously described [8], [9]. Briefly, after a centrifugation and filtration step using 0.22 µM filters, the eluate was subjected to DNase I treatment (100 U/200 µl sample). The resulting virion-enriched samples were subjected to a viral RNA extraction using the QIAamp Viral RNA Mini Kit (Qiagen). Forty units of Protector RNase inhibitor (Roche) were added to the eluted RNA, and RNA quality was checked using a Bioanalyzer 2100 (Agilent Technologies). The random first- and second strand cDNA synthesis was performed with the primer FR26RV-N (5′GCC GGA GCT CTG CAG ATA TCN NNN NN 3′) and primer FR20RV (5′-GCC GGA GCT CTG CAG ATA TC-3′) was used in subsequent PCR to amplify the resulting cDNA. After visualisation of the random amplified DNA fragments on a 1% agarose gel, the fragments between 200 and 1000 bp were excised, purified and quantified using a Nanodrop spectrophotometer (Nanodrop Technologies). Five micrograms of each size selected (200–1000 bp) and purified random amplified sample were sequenced on a GS FLX+ (Roche, Mannheim, Germany) by the Genomics Core of the University Hospital (University of Leuven, Belgium) using multiplex identifier (MID) identification during library preparation and their standard procedures using GS FLX Titanium series reagents (Roche, Mannheim, Germany). The DNA fragmentation step by nebulization was skipped and the intention was to obtain 30000–40000 reads per library.

### Metagenomic analysis

As an additional control to exclude reads originating from potential DNA contamination during the library preparation steps, only reads containing the SISPA primer sequence were included in the assembly. Subsequently, the SISPA primer sequences plus additional six bases were trimmed off the reads. By using a combination of BLAST [17] and sequence mapping with the 454 reference mapper application (version 2.6; Roche), contigs (i.e. sets of overlapping sequence reads) and reads were classified into different taxa.

### Reference assembly

To map the obtained raw sequence data to the genome of Schmallenberg virus, the complete coding sequence of SBV isolate BH80/11-4 (accession codes HE649912–HE649914) was used as reference genome in the reference assemblies of our different field samples using SeqMan NGen® version 3 (DNASTAR, Madison, WI, USA). The reads were first trimmed to remove primer sequences (including the primer-encoded random N positions) as well as low quality ends. Standard assembling and filtering parameters were used, except for a reduced minimum match percentage. The partial sequence information was made accessible through GenBank accession numbers JQ861686–JQ861692, except for fragments that were less than 200 bp in length due to the minimum fragment length requirements dictated by GenBank.

### Variability analysis

The obtained partial sequence information of SBV was compared to the sequence of isolate BH80/11-4 (accession codes HE649912–HE649914). Single nucleotide polymorphisms (SNP's) were identified using SeqMan Pro version 9 (DNASTAR, Madison, WI, USA) and are listed in Table 3. Only sequence differences where we had at least 2 sequence reads were included. Observed sequence variants at a position with more than 2 reads and/or with reads in both cDNA and complementary sense and/or with reads having different start positions were assigned a high support (Table 3).
